# Lymphangiogenesis in Gastric Cancer regulated through Akt/mTOR-VEGF-C/VEGF-D axis

**DOI:** 10.1186/s12885-015-1109-0

**Published:** 2015-03-07

**Authors:** Hongxia Chen, Runnian Guan, Yupeng Lei, Jianyong Chen, Qi Ge, Xiaoshen Zhang, Ruoxu Dou, Hongyuan Chen, Hao Liu, Xiaolong Qi, Xiaodong Zhou, Changyan Chen

**Affiliations:** 1 Department of Gastroenterology, The First Affiliated Hospital of Nanchang University, 17 Yongwaizheng Street, Nanchang, 330006 China; 2 Department of Gastroenterology, Kaiping Central Hospital, Kaiping, 529300 China; 3 Department of Gastroenterology, Jiangxi Provincial People’s Hospital, Nanchang, 330006 China; 4 Department of Radiation Oncology, Massachusetts General Hospital and Harvard Medical School, Boston, MA USA; 5 Department of Pathogen Biology and Immunology, School of Basic Course, Guangdong Pharmaceutical University, Guangzhou, 510060 China; 6 Center for Drug Discovery, Northeastern University, Boston, MA 02115 USA

**Keywords:** Lymphangiogenesis, Gastric cancer, Akt/mTOR, VEGF

## Abstract

**Background:**

Lymphangiogenesis plays a significant role in metastasis and recurrence of gastric cancer. There is no report yet focusing on the modulation of VEGF pathway and lymphangiogenesis of gastric cancer by targeting Akt/mTOR pathway. This study aims to demonstrate the relationship between Akt/mTOR pathway and VEGF-C/-D in gastric cancer.

**Methods:**

We collected surgically resected gastric adenocarcinoma specimens from 55 consented patients. Immunohistochemistry staining of p-Akt, p-mTOR, VEGF-C, VEGF-D were performed and scored by two independent pathologists. The results were presented as staining intensity and positive staining cell rate. We also measured lymphatic vessel density (LVD) by D2-40 staining. Different dosages of p-Akt inhibitor LY294002 (12.5 μM, 25 μM, 50 μM) and p-mTOR inhibitor Rapamycin (25 nM, 50 nM, 100 nM) were given to gastric cancer cell line SGC-7901 in vitro. The inhibition rate of cell growth was tested by MTT at 24 h, 48 h and 72 h, respectively and protein expressions of Akt, p-Akt, mTOR, p-mTOR, VEGF-C and VEGF-D were examined by Western blot.

**Results:**

The positive staining rates of p-Akt, p-mTOR, VEGF-C and VEGF-D in 55 gastric cancer clinical specimens were 74.54%, 85.45%, 72.73% and 58.18%. p-Akt and p-mTOR were positively correlated with VEGF-C and VEGF-D (p < 0.01). The LVD increased with incremental tendency of staining intensity of p-Akt, p-mTOR, VEGF-C and VEGF-D. LY294002 or Rapamycin significantly suppressed SGC-7901 cell growth and the inhibition rate was dose and time dependent (p < 0.001). In addition, the protein expression of p-Akt and p-mTOR were positively correlated with that of VEGF-C and VEGF-D (p < 0.05).

**Conclusions:**

The level of LVD in gastric cancer specimens was significant higher than that of normal gastric tissue and was positively correlated with p-Akt, p-mTOR, VEGF-C and VEGF-D. Inhibition of p-Akt and p-mTOR, in vitro, decreased tumor cell VEGF-C and VEGF-D significantly. Therefore, we concluded that lymphangiogenesis of gastric cancer might be related to Akt/mTOR-VEGF-C/VEGF-D axis.

## Background

Gastric cancer is one of the most common malignant neoplasms worldwide [1-3]. Common treatment strategies for gastric cancer include surgery, radiotherapy, chemotherapy and targeted therapy [4,5]. Treatment failures include metastasis and recurrence after operation. The main metastatic routes of gastric cancer are direct invasion, vascular metastasis, lymphatic metastasis and enterocoelia metastasis [3]. Among those, lymphatic metastasis is an increasingly important criterion in judging the prognosis of gastric carcinoma and lymphangiogenesis is recognized as a significant predictor for the prognosis of patients with gastric cancer [1,2]. In addition, the stomach cancer lymphatic metastasis is believed to be an earlier event compared to vascular metastasis, happening even in early stage gastric cancer [3,4]. Therefore, the International Union against Cancer took the lymphangiogenesis rate and the metastasis amount of the lymph node into the staging of gastric cancer in evaluating the prognosis [2,5]. Besides, even if lymphadenectomy and intraperitoneal chemotherapy were performed during lymph node negative progression, it also brought the positive effect on improving the 5-year survival rate of gastric cancer [5].

The two established ways of tumor cells entry into the lymprhatic system are invasion of existing lymphatic ducts and induction of lymphangiogenesis [3]. The latter is considered as the major way. In addition, lymphangiogenesis plays a decisive role in metastasis, recurrence and prognosis in early gastric cancer [4]. Thus, studies of lymphangiogenesis have a promising impact on the treatment. VEGF-C and VEGF-D were positively related to solid tumor lymphatic metastasis, lymphatic vessel density (LVD) and depth of tumor invasion and recurrence according to previous studies [6-8]. Besides, Akt/mTOR pathway is also involved in the regulation of lymphangiogenesis and lymphatic metastasis [9-11]. Therefore, the relationship between Akt/mTOR pathway and VEGF pathways is worthy to be elucidated. To our knowledge, there is no report yet focusing on the modulation of VEGF pathway and lymphangiogenesis by targeting Akt/mTOR pathway in gastric cancer. In this study, we aim to demonstrate the relationship between Akt/mTOR pathway and VEGF-C/-D in gastric cancer.

## Methods

### Clinical specimen

Surgical resected gastric adenocarcinoma specimens were collected from 55 patients with gastric cancer from April 2011 to November 2012. All of them received no radiotherapy, chemotherapy or immunotherapy before the operation. The study was approved by ethics committee of the First Affiliated Hospital of Nanchang University and all participants gave written informed consent.

### Immunohistochemistry

All tissue samples were fixed with 10% formalin, routinely embedded in paraffin, cut into 4-μm thick as serial sections, dewaxed with xylene, rehydration with an ethanol gradient and washed with distilled water. Antigen retrieval was performed by submerging sections in antigenic retrieval buffer (0.5 M EDTA), and microwaving for 20 min. Sections were then treated with 3% hydrogen peroxide solution. The samples were blocked with 5% normal goat serum and then incubated overnight at 4°C with the primary antibody: p-Akt (ser473) (Rabbit, 1:100, Cell Signaling Technology, USA), p-mTOR (ser2448) (Rabbit, 1:200, Abcam, USA), VEGF-C (Goat, 1:400, R&D, USA), VEGF-D (Mouse, 1:50, Santa Cruz, USA) and D2-40 (Mouse, 1:100, ZSGB-BIO, China). Then, second antibody was added, and the samples were incubated for 30 min at room temperature and then developed with DAB. 5% normal goat serum was used as a negative control instead of the primary antibody. Immunohistochemistry staining was scored by two independent pathologists. Results were presented in the form of staining intensity (+/++/+++) and positive staining cell rate.

### Lymphatic vessel density

According to Masakau criteria, D2-40 positive staining (single endothelial cell or cell clusters) was marked as positive microlymphatic vessel. The vascular-rich area in para-neoplastic location and tumor center were defined and 5 fields of “hot spots” were randomly counted under high magnification (×400). The average was counted as the LVD.

### Cell survival

Three different dosages (12.5 μM, 25 μM, 50 μM) of Akt activation inhibitor LY294002 (Sigma) or mTOR activation inhibitor (25 nM, 50 nM, 100 nM) Rapamycin (Sigma) were given to gastric cancer cell line SGC-7901. The inhibition rate of cell growth was tested by MTT assay at 24 h, 48 h and 72 h, respectively.

### Western blot

SGC-7901 cells were homogenized in RIPA lysis buffer (10 mM Tris–HCl, pH 7.4, 150 mM NaCl, 600 mM NP-40, 1% Triton X-100, 10% glycerol, 1 mM phenylmethylsulfonyl fluoride, 1 mM sodium fluoride, and 1 mM sodium orthovanadate) and put on ice for 2 h. Protein concentration of each sample was determined by Bio-Rad Protein Assay kit (Bio-Rad, Laboratories, Hercules, CA, USA) according to the instruction of manufacturer. Lysates containing 50 μg of protein were separated by SDS-PAGE and then transferred to nitrocellulose membranes. Nonspecific reactions were blocked for 4 h with TBS-T (50 mM Tris.HCl, pH7.4, 150 mM NaCl, 0.1% Tween 20) containing 5% non-fat dry milk. Then membranes were incubated overnight at 4°C with primary antibody: Akt (Mouse, 1:2000, Cell Signaling Technology, USA), p-Akt (ser473) (Rabbit, 1:2000, Cell Signaling Technology, USA), mTOR (Rabbit, 1:1000, Cell Signaling Technology, USA), p-mTOR (ser2448) (Rabbit, 1:3000, Abcam, USA), VEGF-C (Goat, 1:1000, R&D, USA) and VEGF-D (Mouse, 1:500, Santa Cruz, USA). After being washed with TBS-T containing non-fat dry milk, the membranes were incubated with horseradish peroxidase-conjugated secondary antibodies. The protein blots were visualized by chemiluminescence using ECL (Amersham).

### Statistical analysis

Independent samples T test between two groups and single factor analysis of variance (ANOVA) for multiple comparisons were utilized when the data in accordance with normal distribution and homogeneity of variance. Otherwise, Mann–Whitney test between two groups and Kruskal-Wallis test among multi-groups were conducted. Spearman analysis was used to measure the correlation of measurement and count data. Statistical analyses were performed by SPSS17.0 (SPSS Inc., Chicago, IL, USA). A p-value of < 0.05 was considered statistically significant.

## Results

### Expression of p-Akt, p-mTOR, VEGF-C and VEGF-D in situ

**Figure 1:**
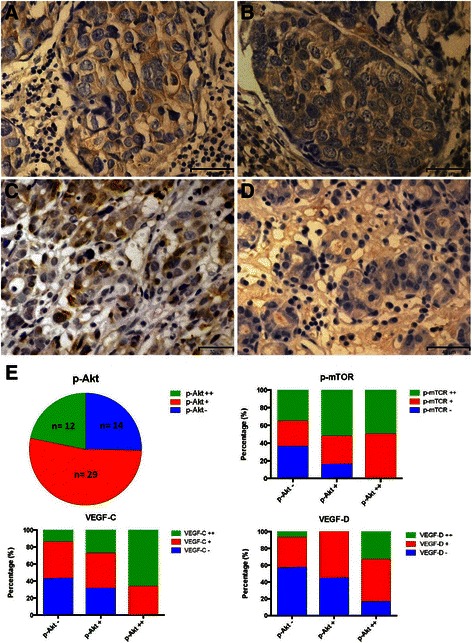
**Immunohistochemistry staining of p-Akt, p-mTOR, VEGF-C and VEGF-D in gastric cancer specimen (×400). A**: p-Akt, **B**: p-mTOR, **C**: VEGF-C, **D**: VEGF-D, **E**: The correlation between p-Akt and p-mTOR/VEGF-C/VEGF-D.

The immunohistochemistry stainings of p-Akt, p-mTOR, VEGF-C and VEGF-D in 55 gastric cancer clinical specimens were shown (Figure 1). The positive staining presented mostly in cytoplasm and a few nuclear stained in p-Akt and p-mTOR. The positivity rates of p-Akt, p-mTOR, VEGF-C and VEGF-D were 74.54%, 85.45%, 72.73% and 58.18% respectively. The quantification of staining intensity was shown in Table 1.

**Table 1:** **Immunohistochemistry staining of p-Akt, p-mTOR, VEGF-C and VEGF-D in gastric cancer specimens**

	Intensity (n)			Positive rate (%)
	-	+	++	
p-Akt	14	29	12	74.54
p-mTOR	8	21	26	85.45
VEGF-C	15	22	18	72.73
VEGF-D	23	27	5	58.18

The correlations of p-Akt and p-mTOR/VEGF-C/VEGF-D, p-mTOR and VEGF-C/VEGF-D were further analyzed. p-Akt expression was positively correlated with p-mTOR (p = 0.001), VEGF-C (p = 0.002) and VEGF-D (p = 0.009) (Figure 1E). Besides, p-mTOR was also significantly positive related to VEGF-C (p < 0.001) and VEGF-D (p = 0.003).

### Lymphatic vessel density

**Figure 2:**
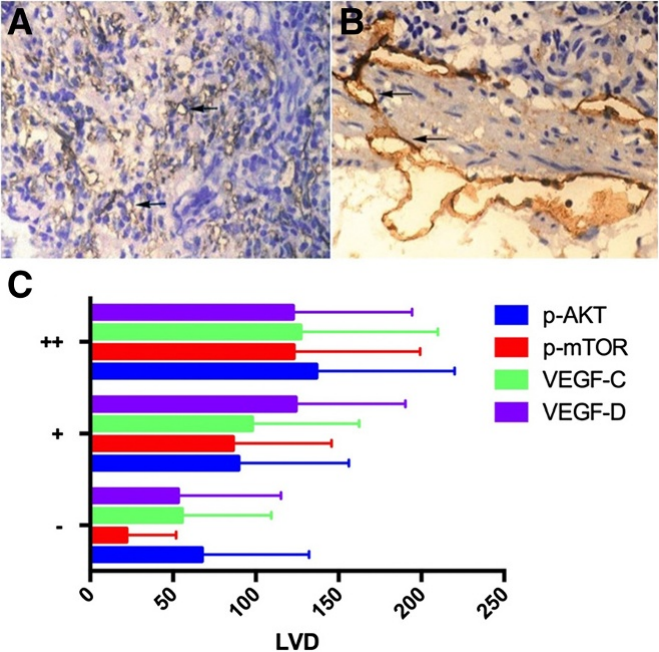
**Immunohistochemistry staining of D2-40 for LVD (×400). A**: Gastric cancer tissue, **B**: Matched normal tissue, **C**: The correlation between LVD and p-Akt/p-mTOR/VEGF-C/VEGF-D. LVD = lymphatic vessel density.

D2-40 staining was used to calculate the LVD for gastric cancer (Figure 2A) and matched normal gastric tissue (Figure 2B). According to the results, D2-40 stained mainly in lymphatic endothelium in scattered or clustered distribution. Thin lymphatic vessel wall was lacking basal layer. The highest LVD was observed at the edge of tumor with a dense cluster-like morphology. In addition, D2-40 was expressed in the submucosa of normal tissue, with a weaker expression in the mucosa or muscle layer as well. The average LVD of gastric carcinoma and normal tissue were 94.18 ± 72.965 vs 23.31 ± 21.569 number/5 high power fields, p < 0.001.

LVD were positively correlated with p-Akt, p-mTOR, VEGF-C and VEGF-D expression per Spearman analysis (p < 0.05). LVD increased with the incremental tendency of staining intensity of p-Akt, p-mTOR, VEGF-C and VEGF-D (Figure 2C).

### Effects of LY294002 and Rapamycin on SGC-7901 cell growth

**Figure 3:**
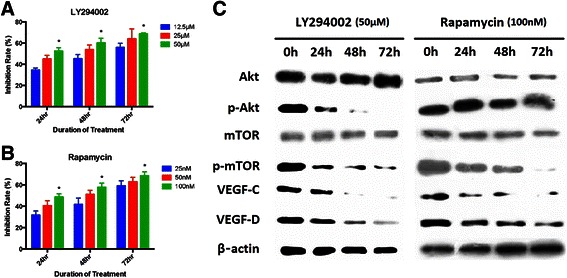
**Inhibition of p-Akt and p-mTOR in SGC-7901 cells by LY294002 and Rapamycin. A**: SGC-7901 cell growth with intervention of different time points and dosages of LY294002, **B**: SGC-7901 cell growth with intervention of different time points and dosages of Rapamycin, **C**: Effect of LY294002 (50 μM) and Rapamycin (100 nM) on Akt/mTOR pathway and VEGF-C/-D by Western blot.

SGC-7901 cells were cultured with three different dosages of LY294002 for 24 h, 48 h and 72 h. According to MTT assay, SGC-7901 cell growth was curbed and the inhibition rate was significantly correlated with LY294002 dosage and action time (p < 0.001) (Figure 3A).

Besides, cells were also cultured with different dosages of Rapamycin for 24 h, 48 h and 72 h, respectively. The inhibition rate of SGC-7901 was also significantly correlated with either Rapamycin dosage or action time (p < 0.001) (Figure 3B). Therefore, both LY294002 and Rapamycin could inhibit the growth of SGC-7901 in either time-dependent or dose-dependent manner.

### Effect of LY294002 on Akt/mTOR pathway and VEGF-C/-D

The protein level of Akt, p-Akt, mTOR, p-mTOR, VEGF-C and VEGF-D in SGC-7901 with LY294002 (50 μM) inhibition were examined by Western blot. Among them, p-Akt, p-mTOR, VEGF-C and VEGF-D were gradually decreased with the prolongation action time of LY294002 (Figure 3C). There was a significant difference when compared with the group without LY294002 (p < 0.05). However, there was no statistical difference for Akt and mTOR (p > 0.05). By further conducting a dual variant relation analysis, p-Akt was positively correlated with p-mTOR/VEGF-C/VEGF-D (p < 0.05) and p-mTOR was also positively related to VEGF-C/VEGF-D (p < 0.05).

### Effect of Rapamycin on mTOR pathway and VEGF-C/-D

The protein level of Akt, p-Akt, mTOR, p-mTOR, VEGF-C and VEGF-D in SGC-7901 with Rapamycin (100 nM) inhibition were examined by Western blot. Among them, p-mTOR, VEGF-C and VEGF-D expression were decreased with the prolongation action time of Rapamycin (Figure 3C). There was a significant difference when compared with the group without Rapamycin (p < 0.05). However, there was no statistical difference for Akt, p-Akt and mTOR (p > 0.05). By further conducting a dual variant relation analysis, p-mTOR was positively correlated with VEGF-C and VEGF-D (p < 0.05).

## Discussion

Akt/mTOR signaling pathway is an important intracellular pathway, which responds to cytokine signals, and is proved to play a major role in cell growth, metabolism and apoptosis [12,13]. Akt/mTOR pathway involves in various pathological mechanisms of colorectal cancer, gastric cancer, liver cancer, breast cancer and uterine cancer etc. [12-14]. Our previous study confirmed that p-Akt involved in angiogenesis of gastric adenocarcinoma and Akt activation may contribute to angiogenesis via VEGF-A up-regulation [15]. In this study, the positive staining rates of p-Akt and p-mTOR in 55 cases of gastric cancer were 74.54% and 85.45% respectively. Besides, p-Akt was positively correlated with p-mTOR (p < 0.01). This result was consistent with previous studies and confirmed the significant role of Akt/mTOR pathway in gastric cancer. The clinical prognosis of gastric cancer is also proposed to be associated with Akt pathway and VEGF pathway. For instance, *Murakami et al.* suggested that the prognosis of gastric cancer patients without p-Akt expression was always better than p-Akt positive ones, and the 5-year survival rates were 68% and 35% for patients with T3 or T4 stage tumors, respectively [16]. Besides, the Akt encoding gene *PI3CA* of upstream protein PI3K was associated with disease-free survival [17]. In addition, *Wang et al.* indicated that VEGF-C, VEGF-D and LVD of gastric cancer were related to tumor size and overall survival according to a study in 123 cases [18,19]. The correlation between VEGF-C/LVD and prognosis in tumor differentiation, T stage, lymph node metastasis, and distant metastasis were also confirmed [20]. Another study further suggested using the combination of VEGF-C/-D and CA199 to predict lymphatic vessel metastasis [21].

Given that Akt/mTOR pathway and VEGF-C/-D play a vital role in lymphatic metastasis and prognosis, our study aims to explore the relationship between two pathways and further promote the mechanism of lymphangiogenesis and metastasis. According to the results of staining *in situ*, p-Akt and p-mTOR were positively correlated with VEGF-C and VEGF-D (p < 0.01). In terms of LVD, D2-40 level of gastric carcinoma and normal tissues were examined at the same time. LVD in gastric cancer was four times higher than that of normal tissue. Besides, LVD was significantly correlated with p-Akt, p-mTOR, VEGF-C and VEGF-D (p < 0.05). Notably, LVD as well as VEGF-C/-D was obviously lower in patients with negative expression of p-Akt and p-mTOR than positive ones. It suggested that both Akt/mTOR pathway and VEGF pathway involved in lymphangiogenesis and lymphatic vessel metastasis in gastric cancer. Similar results were also confirmed by other studies. *Yu et al.* suggested that p-mTOR were highly consistent with lymph node metastasis and could be an independent predictor of lymphatic metastasis and prognosis of gastric cancer [22]. Besides, *Onogawa et al.* indicated that VEGF-C was highly correlated with lymphatic metastasis according to a study conducted in 140 cases of early gastric cancer [23]. VEGF-C as well as VEGF-D was also regarded as a predictor of lymphatic metastasis in patients with early gastric cancer [24]. In the study, we further confirmed the relationship of Akt/mTOR, VEGF-C/-D and LVD in gastric cancer *in situ*.

LY294002, as an Akt activation inhibitor, is confirmed with the anti-tumor effect and promoted the apoptosis of gastric cancer *in vitro* and *in vivo* [25]. Rapamycin known as an mTOR activation inhibitor is clinically used for its anti-tumor effect and immunomodulation [26]. The results of our study further verified that LY294002 and Rapamycin could efficiently inhibit the growth of SGC-7901 cells. Besides, LY294002 at 50 μM) and Rapamycin at 100 nM had the most effective inhibition rate of (52.53 ± 3.19) % and (48.72 ± 3.06) % at 24 h. When prolonging to 72 h, the inhibition rates were (68.76 ± 1.00) % and (68.50 ± 3.77) % respectively. Then, we observed the influence of phosphorylation inhibition of Akt and mTOR on lymphatic factors, VEGF-C and VEGF-D. High concentration of LY294002 (50 μM) and Rapamycin (100 nM) were used for the intervention study. According to the results, Rapamycin suppressed the expression of p-mTOR and LY294002 efficiently inhibited the expression of p-Akt as well as p-mTOR. It is noted that both inhibitors could efficiently inhibit the expression of VEGF-C/-D. Besides, the down-regulation of p-Akt/p-mTOR showed a high consistency with that of VEGF-C/-D. Therefore, Akt/mTOR pathway was proposed to be closely related to VEGF-C/-D. The similar results were also previously reported in other tumor models. For example, *Zhang et al.* decreased the insulin receptor by short hairpin RNA in cancer cell lines, LCC6 and T47D. The cell proliferation, angiogenesis, lymphangiogenesis and metastasis were also inhibited due to the down-regulation of p-Akt and inactivation of VEGF-D [27]. Besides, mTOR inhibition could suppress the expression of VEGF-C and effectively reduce lymphangiogenesis in pancreatic cancer mouse model [11]. In another study, VEGF-D was degraded by Honokiol to inhibit lymphangiogenesis of lung cancer xenograft [28]. IL-6 could also regulate VEGF-C at mRNA level through Akt/mTOR signaling pathway, which effectively decreased the lymphangiogenesis and metastasis in oral squamous cell carcinoma [29].

## Conclusion

The level of LVD in gastric cancer specimens was significant higher than that of normal gastric tissue and positively correlated with immunohistochemistry staining of p-Akt, p-mTOR, VEGF-C and VEGF-D. With the inhibition of p-Akt and p-mTOR in vitro, VEGF-C and VEGF-D were also significantly decreased. Therefore, we propose that lymphangiogenesis of gastric cancer might be efficiently modulated through Akt/mTOR-VEGF-C/VEGF-D axis.

## Abbreviation

LVD: Lymphatic vessel density
